# The Role of Contrast-Enhanced Harmonic Endoscopic Ultrasound in Interventional Endoscopic Ultrasound

**DOI:** 10.3390/medicina57101085

**Published:** 2021-10-11

**Authors:** Cecilia Binda, Chiara Coluccio, Gianmarco Marocchi, Monica Sbrancia, Carlo Fabbri

**Affiliations:** 1 Gastroenterology and Digestive Endoscopy Unit, Forlì-Cesena Hospitals, AUSL Romagna, 47121 Forlì, Italy; chiara.coluccio@auslromagna.it (C.C.); gianmarco.marocchi2@gmail.com (G.M.); monica.sbrancia@auslromagna.it (M.S.); carlo.fabbri@auslromagna.it (C.F.); 2 Department of Medical and Surgical Sciences-DIMEC, Alma Mater Studiorum University of Bologna, 40138 Bologna, Italy

**Keywords:** endoscopic ultrasound, contrast enhancement, contrast-enhanced EUS, interventional EUS, EUS-guided drainage, fine-needle aspiration, tumor ablation, gallbladder drainage, pancreatic fluid collection

## Abstract

Over the last decades, contrast-enhanced harmonic endoscopic ultrasound (CH-EUS) has emerged as an important diagnostic tool for the diagnosis and differentiation of several gastrointestinal diseases. The key advantage of CH-EUS is that the influx and washout of contrast in the target lesion can be observed in real time, accurately depicting microvasculature. CH-EUS is established as an evidence-based technique complementary to B-mode EUS to differentiate solid appearing structures, to characterize mass lesions, and to improve the staging of gastrointestinal and pancreatobiliary cancer. In the last few years, interest has increased in the use of CH-EUS in interventional procedures such as tissue acquisition, tumor ablation, biliary drainage, and the management of pancreatic fluid collections. The aim of this narrative review is to evaluate the available evidence and future expectations of CH-EUS in interventional EUS.

## 1. Introduction

Over the last decades, the development of contrast-enhanced harmonic endoscopic ultrasound (CH-EUS) has emerged as an important diagnostic tool for the diagnosis and differentiation of several gastrointestinal diseases [1,2,3,4].

From the introduction of the first one (Levovist (Bayer Schering Pharma, Berlin, Germany)), several second-generation ultrasound contrast agents have been introduced on the market, namely Sonazoid (Daiichi-Sankyo, Tokyo, Japan; GE Healthcare, Milwaukee, WI, USA), SonoVue (Bracco SpA, Milan, Italy), and Definity (Lantheus Medical Imaging, Billerica, MA, USA), which are composed of gases (perfluorobutane, sulfur hexafluoride, and perflutren, respectively) with a phospholipid or lipid shell. These new agents are more suitable for small transducers and resonate with a lower acoustic power [5].

The key advantage of CH-EUS is that the influx and washout of contrast in the target lesion can be observed in real time, accurately depicting microvasculature. Thus, CH-EUS is established as an evidence-based technique complementary to B-mode EUS to differentiate solid appearing structures, to characterize mass lesions, to improve the staging of gastrointestinal and pancreatobiliary cancer, and to real-time guide diagnostic EUS [6]. Indeed, its main field of application, with high-quality level of evidence, is the differential diagnosis between benign and malignant lesions, especially for pancreatic ones [4].

However, despite its well-known diagnostic role, over the years, interest has increased in the use of CH-EUS in interventional procedures. Several studies have explored its potential benefit in EUS-guided tissue acquisition, in the management of pancreatic fluid collection (PFC), biliary and pancreatic duct drainage, drainage of gallbladder, celiac plexus neurolysis/blockage, drainage of mediastinal and intra-abdominal abscesses and collections, and in targeted cancer chemotherapy and radiotherapy. Moreover, guidelines on CH-EUS have been recently published from the Asian Federation of Societies for Ultrasound in Medicine and Biology [7], providing evidence-based information on technical aspects and indications. In parallel to the widespread of interventional EUS-guided procedures, indeed, CH-EUS could represent an important tool to delineate real-time vascular perfusion and to enhance imaging, in order to have a proper guidance and reduce potential complications.

The aim of this review is to evaluate the available evidence and future expectations of CH-EUS in interventional EUS. We conducted a review of the latest available English-language literature through MEDLINE using the PubMed interface. The following search terms were used: Endoscopic Ultrasound, contrast enhancement, contrast-enhanced EUS, interventional EUS, fine-needle aspiration, tumor ablation, gallbladder drainage, biliary drainage, and pancreatic fluid collection.

## 2. CH-EUS and Tissue Acquisition

Some years far from its introduction, CH-EUS is now considered an extremely useful tool in the characterization of gastrointestinal lesions. As far as CH-EUS-guided tissue acquisition is concerned, the majority of the published studies have evaluated its role in solid pancreatic lesions. Some authors have initially investigated if lesion enhancement during CH-EUS could detect pancreatic adenocarcinoma better than EUS-guided fine needle aspiration (FNA) [8], while others have wondered if CH-EUS could improve the diagnostic yield of EUS-FNA. Kitano et al. [9] showed that the sensitivity in the diagnosis of pancreatic adenocarcinoma increased from 92.2% to 100% combining EUS-FNA histological diagnosis and CH-EUS characteristics.

**Figure 1:**
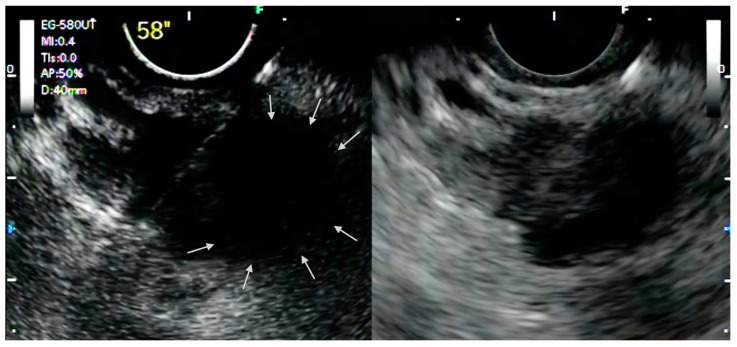
CH-EUS-guided tissue acquisition. View of a CH-EUS-guided FNB of a solid pancreatic lesion with the needle directed to avoid the area without enhancement (arrows).

The ability of CH-EUS to depict the macro- and micro-vascularization of solid lesions in a real-time manner allows the detection of avascular areas, which represent fibrotic or necrotic areas that harbor poor diagnostic potential and that should be avoided during tissue acquisition. Indeed, it has been demonstrated that EUS-FNA has lower sensitivity for pancreatic adenocarcinoma with avascular areas on CH-EUS [10]. In this perspective, it was postulated that CH-EUS could be used to guide EUS-FNA (Figure 1).

In 2015, Sugimoto et al. [11] were the first to compare CH-EUS-guided FNA with conventional EUS-FNA in a prospective randomized trial. Forty patients with solid pancreatic lesions were randomized to receive standard EUS-FNA (20 patients) or CH-EUS evaluation before EUS-FNA in order to direct the biopsy toward the enhancing areas of the lesion. Although the sensitivity and the accuracy were not different between the two groups, the CH-EUS group required a lower number of passes to reach a diagnosis compared to conventional EUS-FNA group (60% of diagnosis with a single pass versus 25%, respectively).

A prospective cohort study by Itonaga et al. [12] showed that in 93 patients with solid pancreatic lesions undergoing consecutively EUS-FNA and CH-EUS-guided FNA (first and second pass, respectively), the adequate sampling rate and the sensitivity were significantly higher for CH-EUS-guided FNA when a non-enhancing area or a homogeneous area was present within a hypo-enhancing lesion detected on CH-EUS, which was respectively indicative of necrosis or peri-tumoral inflammation.

Recently, two randomized trials failed to demonstrate the superiority of CH-EUS-guided FNA over standard EUS-FNA. Seicean et al. [13] evaluated 148 patients with solid pancreatic masses where two passes with a 22-G standard FNA needle were done with EUS-FNA or CH-EUS-guided FNA as first in random order. The authors reported no statically significant differences in diagnostic performance between samples obtained with standard EUS-FNA or when guided by CH-EUS. These results are in line with another randomized trial by Cho et al. [14] on 240 patients (120 patients for CH-EUS-guided FNA group and 120 for the standard EUS-FNA group). In this study, sensitivity after the first needle pass was higher in the CH-EUS group than that in the conventional EUS group, but the difference disappeared after repeating the second needle pass.

Although there is evidence for solid pancreatic masses, the role of CH-EUS for the guidance of FNA in other conditions still needs to be investigated. It is known that the presence of underlying chronic pancreatitis or the presence of a previously placed biliary stent could reduce the diagnostic yield of EUS-guided tissue acquisition. In these settings, CH-EUS was demonstrated to improve the detection of pancreatic lesions, helping to delineate the margins of a suspected lesion [15]. However, there is still little evidence, and larger dedicated studies are needed.

Moreover, the application of CH-EUS for FNA beyond the pancreatic diseases has been addressed in a study by Oh et al. [16] on 30 patients in which CH-EUS was used to detect small hepatic masses identified on cross-sectional imaging that were not suitable for percutaneous abdominal ultrasound (US)-guided biopsy. Only 73% of the lesions were visible on B-mode EUS; contrast enhancement, highlighting the distinction between the target lesion and the surrounding liver parenchyma, allowed the identification of 96.7% of the lesions and the tissue sampling with EUS-FNA with a sensitivity, specificity, and diagnostic accuracy of 85.7%, 100%, and 86.7%, respectively.

In conclusion, CH-EUS has been evaluated as an image-enhancing method that ameliorates the performance of EUS-guided tissue acquisition, above all for pancreatic diseases, leading to promising results downsized by the most recent studies. Rather than being constantly applied, the use of CH-EUS for the guidance of EUS-FNA may be reserved to selected cases, especially when the probability of conventional EUS-FNA failure seems to be higher.

## 3. CH-EUS and Tumor Ablation

EUS-guided tumor ablation is an emerging treatment modality initially introduced for malignant pancreatic lesions unsuitable for surgery [17]. The ability of CH-EUS to delineate tumor perfusion dynamics in a real-time manner and to detect enhancing lesions poorly visible on B-mode EUS is supposed to be useful in performing EUS-guided tumor ablation.

To date, only one study and a few case reports reported the use CH-EUS in the field of tumor ablation. Choi et al. described the use of CH-EUS for the guidance and monitoring of EUS-guided radiofrequency ablation (RFA) of solid abdominal tumors [18]. Nineteen patients (13 pancreatic neuroendocrine tumors, 2 solid pseudopapillary neoplasm, 1 pancreatic insulinoma, 2 adrenal adenomas, and 1 adrenal metastasis from hepatocellular carcinoma) underwent EUS-guided RFA preceded by CH-EUS evaluation. Early treatment response was assessed at 5 and 7 days. CH-EUS showed an absence of enhancement in 7 cases and the presence of residual enhancing foci in 12 cases, indicating the complete response or presence of viable tumor, respectively. In those cases of residual tumors, additional RFA sessions were performed. At 1-year follow-up, a complete response was achieved in 68.4% of cases with a median of two RFA sessions. CH-EUS showed the advantage of performing the assessment of early therapeutic response and the identification of residual viable lesions to target in additional RFA sessions. Two case reports [19,20] described the use of EUS-guided tumor ablation for the treatment of a perianastomotic colorectal cancer metastasis using RFA and the treatment of a hepatocellular carcinoma with ethanol injection. In both cases, CH-EUS was used after ablation to confirm the success of the procedure and to exclude the presence of remnant neoplastic tissue.

Concluding, first experiences with the addition of CH-EUS to EUS-guided tumor ablation have showed interesting results, as contrast enhancement of intratumoral vessels gives fundamental information both on the results of the ablation and on the potential residual neoplastic tissue to target in retreatment. Further studies are needed in this setting to confirm this promising results.

## 4. CH-EUS and Hepatobiliary Interventions

EUS is a well-recognized diagnostic and therapeutic modality in the treatment of biliary diseases, playing over the last decade a crucial role in therapeutic management in this field. However, there are still limited data regarding the role of CH-EUS in biliary diseases; most of them focused on the detection and differential diagnosis of malignancies of the gallbladder.

Indeed, it has been shown that CH-EUS following standard EUS can improve the diagnostic accuracy of gallbladder diseases, especially for the differential diagnosis of benign and malignant lesions, with an increased sensitivity from 82% to 100% when combining both modalities [21].

Researchers have also proposed a perfusion and a vessel enhancement pattern classification of gallbladder wall thickening, concluding that irregular vessel pattern and perfusion defect indicates malignant lesions [22,23].

While the application of CH-EUS as a diagnostic tool in gallbladder thickening is well established, there are still limited data about its role in interventional gallbladder EUS procedures. Transmural EUS-guided gallbladder drainage (EUS-GBD) is a safe and effective interventional endoscopic option for the treatment of patients with acute cholecystitis and considered at high risk for cholecystectomy. Its clinical application is currently endorsed by the Tokyo Guidelines, which introduced EUS-GBD in the treatment pathway, especially for fragile patients affected by grade 2 and 3 of acute cholecystitis [24,25].

Firstly described in 2007 [26], in the last decade, EUS-GBD has gained popularity progressively, in particular after the introduction of specific lumen apposing metal stents (LAMS), reaching optimal technical and clinical success rates that rank respectively in the range of 93–97% and 94–98% [27] and representing nowadays an interesting alternative to endoscopic transpapillary gallbladder drainage (ET-GBD), which is preferred to percutaneous trans-hepatic gallbladder drainage (PT-GBD) when surgery is not considered an option [28].

**Figure 2:**
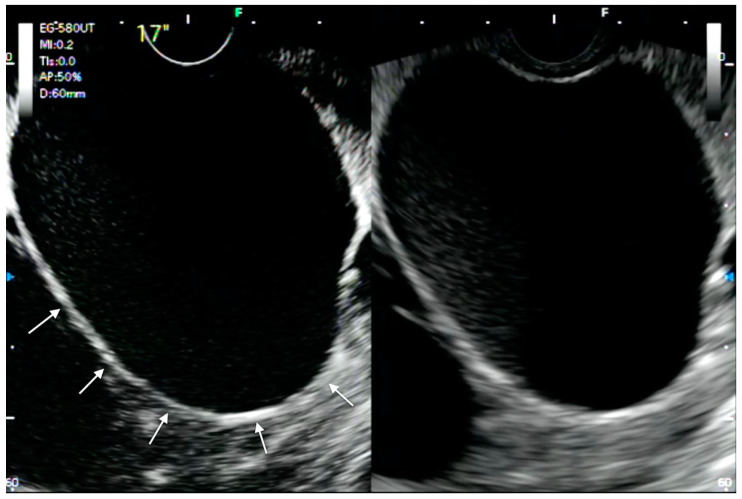
CH-EUS in hepatobiliary evaluation. View of gallbladder wall enhancement (arrows) at CH-EUS evaluation of a hydropic gallbladder.

Concerning EUS-GBD technical aspects, the first step is a diagnostic EUS examination to assess gallbladder features, such as dimensions, wall integrity in order to rule out gangrenous cholecystitis, interposed vessels, and other structures surrounding the intended needle path (Figure 2). The ability of CH-EUS to enhance the micro- and macro-vascularization of a gallbladder wall in real time allows the detection of vessels and avascular areas that could determine intraprocedural complications such as bleeding, perforation, and stent dislodgement. In fact, despite promising results, EUS-GBD is still far from being a perfect strategy, with an adverse event rate of 7–21%. In this scenario, it is crucial to identify patients at different risk of successful outcome. The achievement of technical and clinical successes, as well as complications related to the procedure, would depend on clinical and gallbladder features. Although patients’ features have been assessed for a possible predictor of clinical outcome [29], data are lacking on the impact of morphological features of the gallbladder on technical and clinical outcomes. The absence of enhancement in the gallbladder wall on contrast-enhanced ultrasound (CEUS) evaluation has been reported by Ripolles et al. to predict the presence of gangrenous cholecystitis with a sensitivity of 85–91% and a specificity of 67.5–84.8% [30]. Similarly, evaluation of gallbladder wall enhancement could be easily achieved with CH-EUS, helping to rule out complicated acute cholecystitis or other variables that may impact on EUS-GBD outcomes.

Moreover, EUS to date has played a pivotal role in the diagnosis and therapeutic paradigm of malignant distal biliary obstruction (MBO). Evidence on EUS-guided biliary decompression for the relief of jaundice in this setting of patients, both after failure of endoscopic retrograde cholangiopancreatography (ERCP) or as primary approach has largely increased in recent years [31,32,33,34].

All the studies reported a high level of efficacy and very good safety profile of EUS-guided biliary drainage, although most of the data come from tertiary referral centers [34].

As for EUS-GBD, few data are available on the usefulness of CH-EUS on this setting, although the use of contrast enhancement agents may be helpful in optimizing EUS examination in order to safely perform EUS-guided drainage, reducing the risk of intraprocedural adverse events.

In 2017, Minaga et al. reported a case of MBO in which the common bile duct (CBD) was poorly visible at B-mode evaluation due the presence of a large amount of hypo-echoic material into the lumen. Therefore, in order to exclude the presence of active bleeding into the CBD and to have a better delineation of the CBD and the surrounding tissues, a CH-EUS was performed, allowing a better identification of the CBD and the ability to safely perform an EUS-guided choledochoduodenostomy (EUS-CDS) [35]. Similarly, Tamura et al. [36] reported the use of CH-EUS in five patients with MBO due to bile duct cancer who underwent EUS-guided biliary drainage through an intrahepatic or extrahepatic bile duct approach (respectively three and two patients). The identification of the bile ducts on B-mode EUS was hampered due to the presence of debris and neoplastic tissue; thus, CH-EUS was used to differentiate between them and to clarify the border with the surrounding hepatic parenchyma, allowing obtaining technical and clinical success in all cases.

In conclusion, CH-EUS could be used as an enhancing method in interventional biliary EUS procedures, in particular in EUS-GBD, adding information for better decision making in the management both of acute cholecystitis and MBO, allowing the identification of morphological predictors both of technical success rate and of potential adverse events.

## 5. CH-EUS and Pancreatic Fluid Collections

PFCs are one of the main local complications of acute pancreatitis, and the literature is counting on a continuous understanding of their morphology and technical improvements to handle them. Thanks to the widespread techniques and the introduction of dedicated devices on the market, EUS has become an essential part of both the diagnostic and therapeutic work-out of PFC [37,38,39].

In the last decades, we have perceived a change of paradigm in management of PFC, in parallel to the wide spread of both diagnostic and interventional EUS, allowing an effective and less invasive approach than other conventional treatment.

Over the years, EUS has gained a pivotal role both for the assessment and the treatment of PFC [40]. Thanks to its high detailed spatial resolution, indeed, EUS provides substantial information on the content of PFC, distinguishing among liquid collections, namely pseudocyst (PCs) and walled-off pancreatic necrosis (WOPN), and on the presence of a mature capsule; the correct assessment of the morphology and content of the PFC is to date essential for the choice of the best treatment option [37,38].

EUS has led a better characterization of PFC, especially a better definition of the content of the collections, providing additional, and perhaps more accurate (compared with other radiological techniques such as CT-scan) assessment of solid component within the collections [41]. A study by Rana et al., comparing magnetic resonance (MRI), EUS, and US showed that MRI underestimated the amount of necrosis in patients with <40% solid debris, comparing to EUS and US. US showed an accuracy comparable to EUS and MRI for the evaluation of necrotic collections, although limitations were related to the suboptimal detection of collections and the inability to characterize collections with high solid content or air. Moreover, in the same study, EUS was more accurate for the diagnosis of venous collaterals around the collection, which was important in order to reduce the risk of bleeding due to inadvertent puncture during drainage [42].

Moreover, it has been reported that thanks to its accuracy in characterizing PFC, EUS performed prior to intervention can modify the management in up to one-third of patients because of a change of diagnosis or identification of anatomical and vascular factors precluding endoscopic management [43,44].

**Figure 3:**
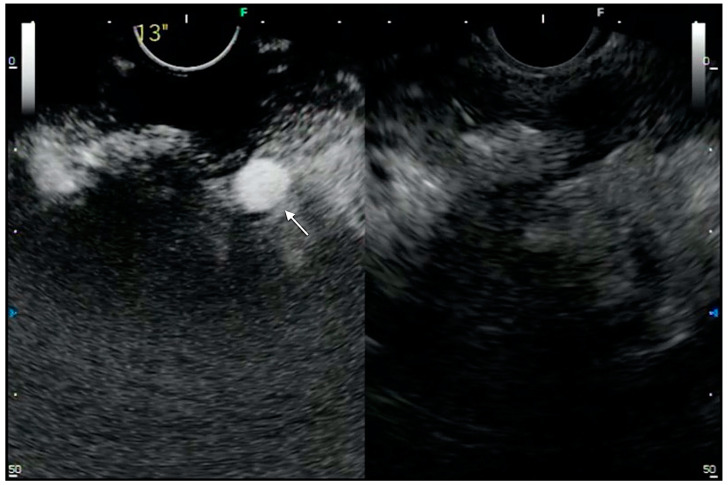
CH-EUS in PFC evaluation. View of splenic artery (arrow) within a walled-off pancreatic necrosis visualized using CH-EUS.

Therefore, in the setting of PFC, CH-EUS could provide a deeper definition of the collection, the content, and vascular complication (e.g., pseudoaneurysms, splenic vein thrombosis, etc.) that could potentially increase the risks of adverse events of the endoscopic treatment (Figure 3).

In 2017, Minaga et al. reported a case of EUS-guided drainage of an infected WOPN under CH-EUS guidance. There is an inability to depict the target lesion and its margins in B-mode due to heterogeneous echogenicity; thus, a CH-EUS was performed to enhance the contrast between the targeted WOPN and the surrounding tissues, enabling the assessment of the microvasculature and hemodynamics of the collection in real time and the ability to safely puncture it and perform the EUS-guided drainage [45].

Moreover, in 2011, Badea et al. reported a case of a 33-year-old male with recurrent episodes of acute pancreatitis on alcohol-induced chronic pancreatitis complicated by PC and bleeding from the major papilla. After both abdominal ultrasound and EUS, the use of a contrast agent allowed revealing the presence of a pseudoaneurysm of the splenic artery within the PC. Due to the continuous hemorrhage, the patient underwent surgery, and there was a diagnosis of wirsungorrhagia due to the rupture of the pseudoaneurysm of the splenic artery in the cyst [46].

In conclusion, as outlined previously, CH-EUS can be successfully used for the evaluation and diagnostic and therapeutic work-up of PFC, and contrast enhancement can also be used to help this evaluation, adding more key points toward a better treatment. Finally, the addition of contrast agents to EUS may aid the interpretation of vascularity and its complications, even though the literature on this topic is limited to a few cases.

## 6. Conclusions and Future Perspectives

Contrast-enhanced EUS could be a valid tool for endosonographers that approach interventional EUS-guided procedures. In fact, in parallel to the increasing invasiveness of EUS, it would be useful to have a detailed roadmap that may both increase the accuracy and efficacy of EUS-guided procedures and may help to predict all those conditions that may be associated to an increased risk of complications. Furthermore, inter-observer agreement in CH-EUS has already been reported as satisfactory among endosonographers, even between experienced and non-experienced ones [47], so it is reasonable to expect that this is true also for CH-EUS when applied to interventional procedures, but further studies should address this issue.

In conclusion, the latest evidence suggests that CH-EUS could be a helpful tool to guide selected cases of EUS-guided tissue acquisition, to evaluate the efficacy of EUS-guided tumor ablation and the need for retreatment, and it provides adjunctive information for predicting technical success and potential adverse events in EUS-guided biliary drainage and endoscopic management of PFC.

As little evidence is still available on the role of CH-EUS in interventional EUS, there are many questions that do still have an ambiguous answer and which may be the objects of future studies (Table 1).

## Figures and Tables

**Table 1:** Future perspective box.

Future Perspectives	
CH-EUS AND TISSUE ACQUISITION	✓ Guidance for tissue acquisition in difficult cases (e.g., chronic pancreatitis) ✓ Evaluation of its usefulness with FNB
CH-EUS AND TUMOR ABLATION	✓ Evaluation of the result of the ablation ✓ Target residual tumor tissue for re-treatment
CH-EUS AND BILIARY INTERVENTIONS	✓ Evaluation of gallbladder wall in acute cholecystitis in order to evaluate the presence of morphological predictors (e.g., necrosis) ✓ Better definition of CBD in difficult cases
CH-EUS AND PANCREATIC FLUID COLLECTIONS	✓ Better definition of the PFC ✓ Better definition of vascularity and of potential complication in case of EUS-guided drainage

## Data Availability

Data sharing not applicable.
